# Co-designing ab initio electronic structure methods on a RISC-V vector architecture

**DOI:** 10.12688/openreseurope.18321.1

**Published:** 2024-08-05

**Authors:** Rogeli Grima Torres, Pablo Vizcaíno, Filippo Mantovani, José Julio Gutiérrez Moreno

**Affiliations:** 1 Barcelona Supercomputing Center (BSC), Plaça Eusebi Güell, 1-3, Barcelona, 08034, Spain

**Keywords:** High-performance computing, co-design, ab initio, RISC-V, materials science, eigensolver library, European processor initiative

## Abstract

*Ab initio* electronic structure applications are among the most widely used in High-Performance Computing (HPC), and the eigenvalue problem is often their main computational bottleneck. This article presents our initial efforts in porting these codes to a RISC-V prototype platform leveraging a wide Vector Processing Unit (VPU). Our software tester is based on a mini-app extracted from the ELPA eigensolver library. The user-space Vehave and a RISC-V vector architecture implemented on an FPGA were tested. Metrics from both systems and different vectorisation strategies were extracted, ranging from the most simple and portable one (using autovectorisation and assisting this by fusing loops in the code) to the more complex one (using intrinsics). We observed a progressive reduction in the number of vectorial instructions, executed instructions and computing cycles with the different methodologies, which will lead to a substantial speed-up in the calculations. The obtained outcomes are crucial in advancing the porting of computational materials and molecular science codes to (post)-exascale architectures using RISC-V-based technologies fully developed within the EU. Our evaluation also provides valuable feedback for hardware designers, engineers and compiler developers, making this use case pivotal for co-design efforts.

## Introduction

*Ab initio* electronic structure applications are among the most popular and computationally demanding in High-Performance Computing (HPC). In recent years, developers have put substantial efforts into modularising codes, moving from rather extensive and sometimes monolithic applications toward more structured software. This new design intends to be more easily adapted to incorporate external libraries for some critical or computationally expensive parts of the codes. This adaptation will facilitate the evolution of codes to the (post-)exascale era and to perform efficiently heterogeneous computing systems ^1^.

Eigenvalue problems are key in *ab initio* electronic structure calculations when solving the Schrödinger equation for many-body extended systems. Eigensolvers are often the main computational bottleneck in Density Functional theory (DFT) calculations, taking over 90% of the total computational time in relatively large systems, limiting the size and complexity of the model in practice. On this ground, the ELPA (Eigenvalue soLvers for Petaflop Applications) library is designed to solve the eigenvalue problem efficiently, supporting efficient CPU and GPU performance on all major HPC platforms ^2– 4^. ELPA is employed by some of the most widely-used DFT codes, such as VASP ^5^, Siesta ^6^, Quantum Espresso ^7^, Abinit ^8^, exciting ^9^, FHI-aims ^10^, GPAW ^11^ or CP2K ^12^. The library can be directly implemented within the code or incorporated as a part of wider open-source software library solutions, such as ELSI ^13^ or SIRIUS ^14^.

Exascale computing represents a significant advancement HPC, unlocking unprecedented opportunities to transform materials and molecular modeling, allowing more accurate calculations, complex morphologies and exploration of large data sets to discover novel compounds ^15^. However, this evolution will come with more heterogeneity in computer architectures, incorporating specialised hardware for specific applications ^16^. Therefore, bidirectional efforts in co-design of hardware and software are required from early-developed prototype systems toward an efficient transition to the post-exascale era.

Our paper describes the initial steps to port the ELPA eigensolver to a prototype platform called EPAC-VEC powered by a RISC-V core coupled with a wide vector unit. This architecture is part of EPAC, a collection of RISC-V based accelerators implemented in a test chip fabricated during 2023 at 22nm within the European Processor Initiative (EPI) project. EPI seeks to reinforce Europe’s digital sovereignty by developing high-performance, energy-efficient processors for supercomputers. EPAC-VEC is an extremely relevant architecture for general purpose HPC since it features a vector unit capable of handling vectors of up to 256 double-precision elements (16,384 bits per vector register) ^17^, 32 times larger than current Single Instruction/Multiple Data (SIMD) architectures such as Intel’s AVX512 extension. This long-vector architecture has already been used to accelerate other HPC workloads such as fast fourier transforms (FFT) ^18^, sparse matrix-vector multiplication ^19^, graph processing algorithms ^20^, and computational fluid dynamics (CDF) ^21^. Given the relevance of the eigensolver, optimisations made on ELPA will eventually benefit the wider community and pave the way for the future portability of electronic structure applications to RISC-V architectures. On the other hand, as a cornerstone of the co-design process, the outcomes collected during landmark work are also beneficial in guiding the development of future hardware and compilers.

## Methodology

Our performance analyses were carried using the so-called Software Development Vehicles (SDV) ^22^. This set of platforms, compilers, and analysis tools allow software developers to run applications on early iterations of the hardware, providing constant feedback to architecture design and the compiler development team, which guarantees the possibility of quickly improving the platform design.

The initial executions were performed on Vehave ^22^, a user-space emulator for the RISC-V Instruction Set Architecture (ISA) vector extension. Vehave runs on top of the RISC-V commercial platforms, intercepting the vectorial instructions, decoding them, and emulating the vector extension. The emulator relies on LLVM ^23^ libraries for instruction decoding and generates detailed Paraver ^24^ trace files containing information about each emulated vector instruction. In addition to the Vehave platform, where vectorial instructions are emulated, we also used a field-programmable gate array (FPGA)-based emulation of the EPAC-VEC chip ^22^. This FPGA is used as user-defined reconfigurable hardware platform emulating the EPAC-VEC test chip. The compiler, based on LLVM, supports autovectorisation and provides built-ins for vector instructions. A reference of the vectorial EPI intrinsics can be consulted in this link ^25^.

Given the limitation of a single (emulated) computing core, the early-stage development of the compiler and the limited availability of libraries, this work has been performed using a mini-app extracted from ELPA, representing a small fraction of the code that retains the primary performance-intensive section. Our mini-app was inspired by a broader suite, developed in the NOMAD Center of Excellence ^26^ framework, to drive co-design in *ab initio* electronic structure calculations. More details on the mini-apps development and execution are given in our recent publication ^27^. Our code isolates the *trans_ev_tridi_to_band* subroutine in ELPA (v.2022.05.001), extracted from the two-stage tridiagonalisation ^2^. This method is normally preferred in large problems and when most eigenvectors must be computed. The kernel was selected based on its computationally cost, independence from external functions (Basic Linear Algebra Subroutines (BLAS) library or communications do not dominate the function), and especially to the extensive effort the ELPA developers made to adapt this kernel to use vectorial instruction on different hardware efficiently (i.e., SSE, AVX(2/512), SPARC64 SSE, ARM SVE(128/256/512), BlueGene/(P/Q), NVIDIA, AMD and Intel GPUs). All the code developed within this project, the indications to execute the mini-app on the RISC-V environment and the ELPA checkpoints for matrices with different sizes are accessible from the associated online repositories.

## Results and discussion

Here, we describe the iterative steps toward adapting the ELPA mini-app to run efficiently on a long vector architecture. The ELPA kernels were initially converted from Fortran to C. The C version of the code allowed full compatibility with version 0.7.1 of the EPI LLVM compiler and the execution on the FPGA platform. After that, the first step was compiling the scalar mini-app on a commercial RISC-V core. This was done to verify the code’s compatibility with the RISC-V architecture, that the compiler supports all data structures and code features, and that the instructions are equivalent in both the C and Fortran versions.

Our initial testing on the VPU used the Vehave emulator. While this application does not give access to measuring computing cycles or execution time, the traces provide the number and type of each executed vector instruction, which is valuable insight for studying code regions with vectorisation potential. Based on that information, we have studied, analysed and enabled the vectorisation of ELPA kernels.

The vectorisation was achieved using three different strategies. These are: (i) enabling compiler auto-vectorisation capabilities, (ii) helping the compiler fuse loops, and (iii) vectorising manually using intrinsics. This bottom-up approach offers several possibilities, from the most simple and portable method to the more complex and time-consuming process for the software engineer. The increasing performance is expected to evolve along with the complexity of the solution.

**Figure 1.**
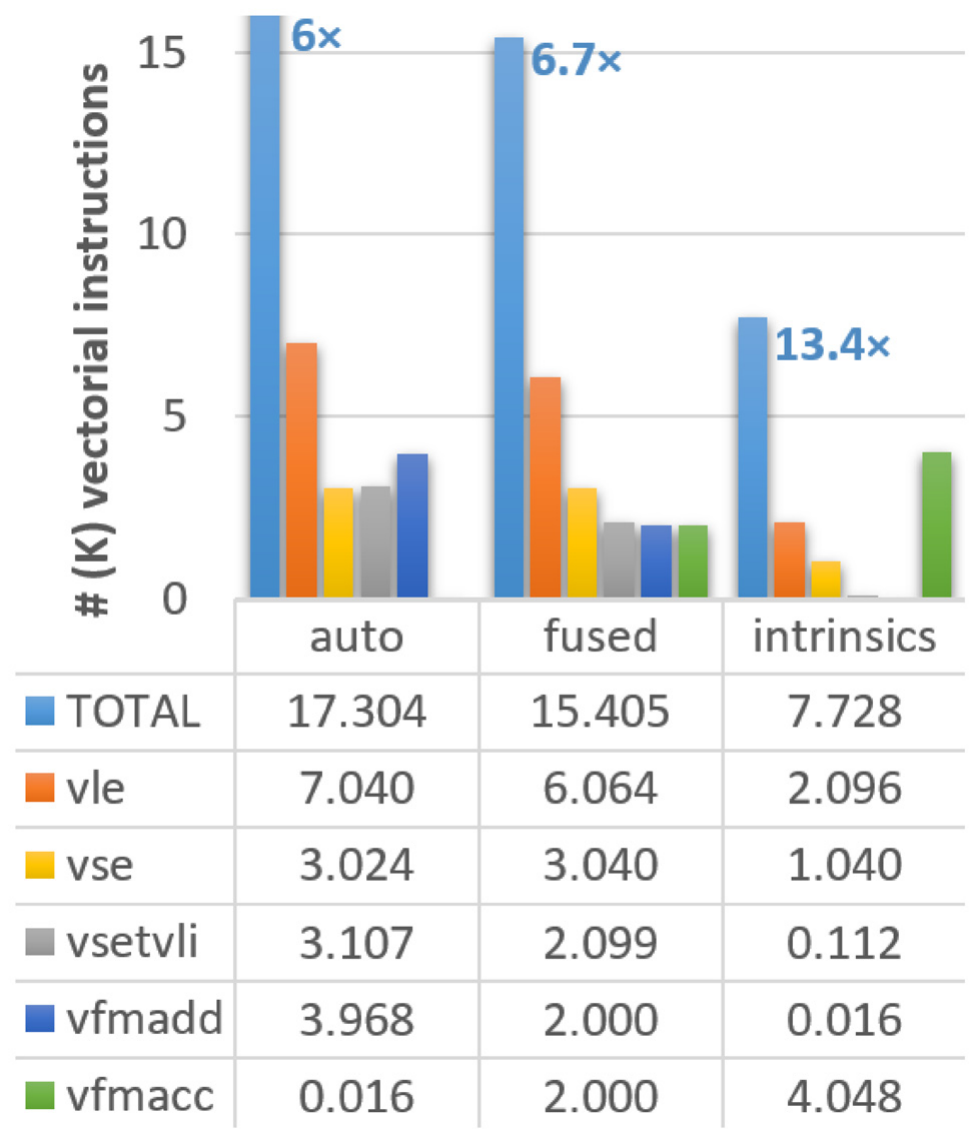
Count of vectorial instructions in the different versions of the ELPA mini-app executed on Vehave. Instructions are distributed by total (light blue bars), 64-bit unit-stride load (vle, orange) and save (vse, orange), settings of vector length (vsetvli, gray), and multiplication-additions (vfmadd in dark blue, vfmacc in green). The reduction in vector instructions of the differently adapted versions compared to the stripped (original) one (which counted a total of 103,784 vector instructions) is indicated over the first bar. Numbers in the graph and table are expressed in thousands.

By leveraging the compiler’s autovectorisation capabilities in the ELPA mini-app’s original version, Vehave performance analyses counted for a total of 103,784 vector instructions. However, the traces show that most of the work is done with a vector length of 48 double-precision elements. This happens because ELPA was designed to divide its Q matrix into 48-element stripes. This implementation is very efficient for exploiting memory locality. However, long-vector architectures are more resistant to memory latency ^28^, and would rather benefit from a long vector length. Therefore, this striped distribution came out to be suboptimal for the VPU. Subsequently, by suppressing the Q-stripes, the algorithm starts leveraging its full vector length (256 elements per vector) without limitations of 48 elements, and the vector instructions are reduced by a factor of 6 *×* compared to the original version (from 103,784 to 17,304 vector instructions). We should note that we have verified that the outcomes from the Fortran and C versions were equivalent at this stage. The number of vector instructions for each code version is presented in Figure 1, where the different instruction types are noted in the caption.

Upon inspection of the compilation output, we still identify several loops in which the compiler is not able to reuse loaded vectors, so memory accesses are replicated in a sub-optimal manner. Therefore, our next strategy consisted of assisting the compiler by identifying loops going through the same variables and combining (fusing) them in the code. This strategy allows us to reduce our memory accesses ( *vle* calls in Figure 1) from 7,040 to 6,064. With these changes, the total number of instructions improves by a factor of 6.7 *×*, an additional 11% with respect to the previous version (from 17,304 to 15,405). Moreover, this strategy is expected to improve the performance of any vectorial compiler. 

Our third and more complex method was using intrinsics, which, in some circumstances, can outperform the simple autovectorisation porting approach. The use of intrinsics allows the expansion of vectorisation, creating a pipeline of vectorial instructions in an outer loop, further reducing the number of memory accesses. This strategy further reduces the vector instructions to 9,499 achieving an improvement of 13.4 *×* compared to the initial version.

However, not all instructions have the same computational cost, so converting from the vectorial instructions counters to computing time (or speed up) may not be straightforward. We observe that our modifications managed to substantially reduce the number of memory accesses (loads ( *vle*) and store ( *vse*)), which are among the most computationally costly instructions. In fact, almost 2/3 of the *vle* and *vse* have been suppressed in the version with intrinsics. On the other hand, the number of fused multiply-add instructions ( *vfmadd* and *vfmacc*) remains almost constant. The settings of vector length ( *vsetvli*) are also progressively reduced; however, their overall contribution to the total number of cycles is almost negligible compared to other instructions. In summary, we can conclude that our results show that all instruction types are being consistently reduced, guaranteeing the improved efficiency of the new implementation.

**Figure 2.**
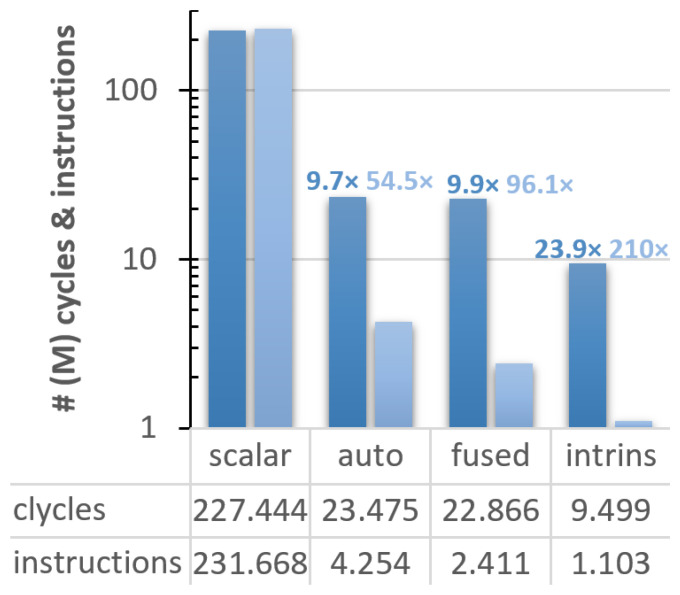
Count of cycles (dark blue bars) and instructions (light blue) for the different vectorised versions of the ELPA mini-app executed on the FPGA system. The speed-up of the differently adapted versions compared to the scalar ones is indicated over the bars, following the same colour code. The y-axis is presented in a logarithmic scale, and all numbers are expressed in millions. PAPI counters were used for these implementations.

After our preliminary study with Vehave, we used an experimental platform to evaluate the RISC-V VPU, composed of an FPGA, and a host-x86 server used to program and communicate with it. In addition to measuring hardware counters such as the number of vector instructions, running on the FPGA allows one to obtain cycle-accurate time measurements. This metric enables a more straight-forward interpretation of how much the code’s efficiency was improved at each implementation. The outcomes of our analyses are presented in Figure 2.

In this case, the scalar version exhibits almost a one-to-one ratio between cycles (227,443,896) and instructions (231,667,638). Compared to the scalar version, the autovectorised one reduces the cycles and instructions by a factor of 9.7 *×* and 54.5 *×*, respectively. The efficiency was further improved in the version with fused loops, obtaining an overall speedup of 9.9 *×* in cycles and 96.1 *×* in instructions, and 23.9 *×* and 210 *×*, respectively, using intrinsics.

## Conclusions

In this short communication, we describe our efforts to inform the implementation of new (post)-exascale HPC systems based on RISC-V. Our porting is centred around the ELPA eigensolver, a library used by many of the most widely-used *ab initio* electronic structure codes. Therefore, our optimisations will eventually benefit the whole community and pave the way for the future portability of electronic structure packages. On the other hand, as a cornerstone of the co-design process, the outcomes collected from our benchmarks also guide the ongoing development of this future HPC hardware and its compilers. Our porting work was done on the RISC-V core with a VPU developed at the Barcelona Supercomputing Center (BSC) within the framework of the European Processor Initiative (EPI) ^29^. The most revolutionary element in the design of this chip is the inclusion of a vector unit capable of handling vectors of up to 256 double-precision elements, compared to, for example, the AVX-512 SIMD extension from Intel that handles up to 8 doubles. Our testing was carried out using the so-called Software Development Vehicles (SDVs), which allowed us to test our software on the most up-to-date version of the hardware, providing continuous feedback to the architects and compiler developers and guaranteeing the overall improvement of the EPI design.

Our manuscript summarises the iterative steps for improving the performance of a complex HPC library leveraging a RISC-V-based VPU prototype. Our tests used a tool called Vehave, a user-space emulator of the RISC-V vector extension, and an FPGA platform, from which the computing cycles and speed-up metrics are obtained. Vectorisation of the kernel was achieved by (i) auto-vectorisation, (ii) fusing similar loops, and (iii) using intrinsics. This progressive approach offers several possibilities, from the most straightforward and portable approaches to the more complicated ones.

The code adaptations guarantee the portability to future hardware with adaptable vector sizes, while we also expect improved performance with other compilers. Moreover, the new v1.0 of the V-extension will allow the compilation of codes in both Fortran and C, so future porting of Fortran code will be more straightforward when the updated hardware is available. We should note that the ELPA library has a patterned file that can be used to create specific kernels for new architectures. Therefore, while we will focus on a specific kernel, porting can be further replicated throughout the library following an analogous procedure. The experience gained provides practical guidance for other codes and architectures. In addition, our mini-app-based model represents a pragmatic, user-friendly approach to facilitate co-design efforts, cooperatively in fine-tuning the software and hardware components. The outcomes of these efforts will contribute significantly to advancing the porting of *ab initio* computational materials and molecular science codes - one of the most relevant families of applications with more users in the HPC community - to (post-)exascale hardware architectures developed in the EU. Furthermore, this evaluation serves as valuable feedback for hardware designers, system integrators and engineers actively involved in compilers for the systems.

## Software availability

All the code developed for this work has been openly stored in Zenodo, https://doi.org/10.5281/zenodo.12665268 ^30^, while the project is also openly available on our institutional BSC Materials Science group GitLab https://gitlab.bsc.es/ material-science/elpa2riscv-public. The code is published under license: GNU General Public License v3.0. The ELPA library is also open-source and can be obtained from https://elpa.mpcdf.mpg.de/. The specification of the RISC-V "V" extension v0.7.1 used in this work can be openly accessed at https://github.com/riscv/riscv-v-spec/releases/tag/0.7.1, under a Creative Commons Attribution 4.0 International Public License. The intrinsics used with the EPI compiler can also be accessed at https://admin.hca.bsc.es/epi/ftp/doc/intrinsics/EPI-0.7/epi-intrinsics.html.
