# Co-designing ab initio electronic structure methods on a RISC-V vector architecture

**DOI:** 10.12688/openreseurope.18321.4

**Published:** 2025-09-09

**Authors:** Rogeli Grima Torres, Pablo Vizcaíno, Filippo Mantovani, José Julio Gutiérrez Moreno

**Affiliations:** 1 Barcelona Supercomputing Center (BSC), Plaça Eusebi Güell, 1-3, Barcelona, 08034, Spain

**Keywords:** High-performance computing, co-design, ab initio, RISC-V, materials science, eigensolver library, European processor initiative

## Abstract

*Ab initio* electronic structure applications are among the most widely used in High-Performance Computing (HPC), and the eigenvalue problem is often their main computational bottleneck. This article presents our initial efforts in porting these codes to a RISC-V prototype platform leveraging a wide Vector Processing Unit (VPU). Our software tester is based on a mini-app extracted from the ELPA eigensolver library. The user-space emulator Vehave and a RISC-V vector architecture implemented on an FPGA were tested. Metrics from both systems and different vectorisation strategies were extracted, ranging from the simplest and most portable one (using autovectorisation and assisting this by fusing loops in the code) to the more complex one (using intrinsics). We observed a progressive reduction in the number of vectorised instructions, executed instructions and computing cycles with the different methodologies, which will lead to a substantial speed-up in the calculations. The obtained outcomes are crucial in advancing the porting of computational materials and molecular science codes to (post)-exascale architectures using RISC-V-based technologies fully developed within the EU. Our evaluation also provides valuable feedback for hardware designers, engineers and compiler developers, making this use case pivotal for co-design efforts.

## Introduction

*Ab initio* electronic structure applications are among the most popular and computationally demanding in High-Performance Computing (HPC). In recent years, developers have put substantial efforts into modularising codes, moving from rather extensive and sometimes monolithic applications toward more structured software. This new design intends to be more adaptable to the incorporation of external libraries for some critical or computationally expensive parts of the codes ^1^. This adaptation will facilitate the evolution of codes to the (post-)exascale era and to perform efficiently on heterogeneous computing systems ^2^.

Eigenvalue problems are key in *ab initio* electronic structure calculations when solving the Schrödinger equation for many-body extended systems. Eigensolvers are often the main computational bottleneck in Density Functional Theory (DFT) calculations. This is due to the cubic scaling growth in computational costs with respect with the problem size, which limits the size and complexity of the model in practice. From our experience as users and developers, and based on extensive performance analyses, we have observed that eigenvalue solvers can consume up to over 90% of the total computational time for relatively large systems. The ELPA (Eigenvalue soLvers for Petaflop Applications) library is designed to solve dense eigenvalue problems (e.g. matrices where most or all elements are non-zero) efficiently, while it is also used as a building block for sparse solvers, supporting efficient CPU and GPU performance on all major HPC platforms ^3– 5^. ELPA is employed by some of the most widely-used DFT codes, such as VASP ^6^, Siesta ^7^, Quantum Espresso ^8^, Abinit ^9^, exciting ^10^, FHI-aims ^11^, GPAW ^12^ and CP2K ^13^. The library can be directly integrated within the code or incorporated as a part of wider open-source software library solutions, such as ELSI ^14^ or SIRIUS ^15^.

Exascale computing represents a significant advancement in HPC, unlocking unprecedented opportunities to transform materials and molecular modeling, allowing more accurate calculations, complex morphologies and exploration of large data sets to discover novel compounds ^16^. However, this evolution will come with more heterogeneity in computer architectures, incorporating specialised hardware for specific applications ^17^. Therefore, bidirectional efforts in co-design of hardware and software are required from early-developed prototype systems toward an efficient transition to the post-exascale era.

Our paper describes the initial steps to port the ELPA eigensolver to a prototype platform called EPAC-VEC powered by a RISC-V core coupled with a wide vector unit. This architecture is part of EPAC, a collection of RISC-V based accelerators implemented in a test chip fabricated during 2023 at 22nm within the European Processor Initiative (EPI) project. EPI seeks to reinforce Europe’s digital sovereignty by developing high-performance, energy-efficient processors for supercomputers. EPAC-VEC is an extremely relevant architecture for general purpose HPC since it features a vector unit capable of handling vectors of up to 256 double-precision elements (16,384 bits per vector register) ^18^, 32 times larger than current Single Instruction/Multiple Data (SIMD) architectures such as Intel’s AVX512 extension. This long-vector architecture has already been used to accelerate other HPC workloads such as Fast Fourier Transforms (FFT) ^19^, sparse matrix-vector multiplication ^20^ or graph processing algorithms ^21^. We have also tested EPAC-VEC for other scientific software applications such as Alya ^22^, a computational fluid dynamic (CFD) code developed in Fortran and we demonstrated speed-ups up to 7× using code modifications that leave the code portable and deliver performance improvements also on Intel x86 architectures (while leveraging AVX512 SIMD extension by Intel). Given the relevance of eigensolvers, optimisations made on ELPA will eventually benefit the wider community and pave the way for the future portability of electronic structure applications to RISC-V architectures. On the other hand, as a cornerstone of the co-design process, the outcomes collected during pioneering works are also beneficial in guiding the development of future hardware and compilers.

## Methodology

Our performance analyses were carried using the so-called Software Development Vehicles (SDV) ^23^. This set of platforms, compilers, and analysis tools allow software developers to run applications on early iterations of the hardware, providing constant feedback to architecture design and the compiler development team, which guarantees the possibility of quickly improving the platform design.

The initial executions were performed on Vehave ^23^, a user-space emulator for the RISC-V Instruction Set Architecture (ISA) vector extension. Vehave runs on top of RISC-V commercial platforms, intercepting the vectorised instructions, decoding them, and emulating the vector extension. The emulator relies on LLVM ^24^ libraries for instruction decoding and generates detailed Paraver ^25^ trace files containing information about each emulated vector instruction. In addition to the Vehave platform, where vector instructions are emulated, we also used a field-programmable gate array (FPGA)-based emulation of the EPAC-VEC chip ^23^. This FPGA is used as a user-defined reconfigurable hardware platform emulating the EPAC-VEC test chip. While emulators are useful for early testing and development, FPGAs enable us to test the design in real-world conditions with accurate timing, ensuring that the system meets performance targets in a hardware environment. The compiler, based on LLVM, supports autovectorisation and provides built-ins for vector instructions. A reference of the vector EPI intrinsics can be consulted online ^26^. The Vehave emulator and the FPGA implementation serve different purposes and offer unique advantages. Vehave allows for quick iteration, debugging, and testing of different configurations without needing to reconfigure hardware. This is particularly useful when exploring architectural optimisations or validating functionalities under varying conditions. On the other hand, the FPGA implementation offers cycle-accurate performance and validates that our design operates as expected when mapped onto a physical implementation.

Given the limitation of a single (emulated) computing core, the early-stage development of the compiler and the limited availability of libraries, this work has been performed using a mini-app extracted from ELPA, representing a small fraction of the code that retains the primary performance-intensive section. Our mini-app was inspired by a broader suite, developed in the NOMAD Center of Excellence ^27^ framework, to drive co-design in *ab initio* electronic structure calculations. More details on the mini-apps development and execution are given in our recent publication ^28^ and its associated repository ( https://gitlab.bsc.es/material-science/nomad-mini-apps-suite). Our code isolates the *trans_ev_tridi_to_band* subroutine in ELPA (v.2022.05.001), extracted from the two-stage tridiagonalisation ^3^. This method is normally preferred in large problems and when most eigenvectors must be computed. The kernel was selected based on its computational cost, independence from external functions (Basic Linear Algebra Subroutines (BLAS) library or communications do not dominate the function), and especially for the extensive effort the ELPA developers made to adapt this kernel to use vector instruction on different hardware efficiently (i.e., SSE, AVX(2/512), SPARC64 SSE, ARM SVE(128/256/512), BlueGene/(P/Q), NVIDIA, AMD and Intel GPUs).

All the code developed within this project, the instructions to execute the mini-app on the RISC-V environment and the ELPA checkpoints (for real double-precision random matrices using the 2-stage method solver with different sizes) are accessible from the associated online repositories. We saved different checkpoints of the application at the entrance of the function *trans_ev_tridi_to_band*. Each checkpoint is labeled with the matrix size, the block size, and the number of eigenvectors that are computed. Note that we have vectorised our code in the direction of the number of eigenvectors and it is very sensitive to this value. For the sake of correctness, we tested all the kernels of our code using Vehave, using every available checkpoint. To test the vector behavior, we focused on our most relevant cases, with large matrix sizes and a number of eigenvectors greater than the vector length. The default checkpoint file is a matrix of 1024 elements, 256 eigenvectors and a blocksize of 32 elements.

## Results and discussion

Here, we describe the iterative steps toward adapting the ELPA mini-app to run efficiently on a long vector architecture. The ELPA kernels were initially converted from Fortran to C. The C version of the code allowed full compatibility with version 0.7.1 of the EPI LLVM compiler and the execution on the FPGA platform. Whereas the binaries produced from the two codes where different, the number and type of vector instructions produced by both versions were proven to be analogous. We should remark that at the time this work was carried out, Vehave used the latest v1.0 implementation of EPI, while the FPGA was on the previous v0.7.1. This new v1.0 of the V-extension allows the compilation of codes in both Fortran and C, while v0.7.1 did not allow Fortran compilations.

After that, the first step was compiling the scalar mini-app on a commercial RISC-V core. This was done to verify the code’s compatibility with the RISC-V architecture, that the compiler supports all data structures and code features, and that the instructions are equivalent in both the C and Fortran versions.

Our initial testing on the VPU used the Vehave emulator. While this application does not give access to measuring computing cycles or execution time, the traces provide the number and type of each executed vector instruction, which is valuable insight for studying code regions with vectorisation potential. Based on that information, we have studied, analysed and enabled the vectorisation of ELPA kernels.

The vectorisation was achieved using three different strategies, starting with the least effort and gradually increasing in complexity. These are: (i) enabling compiler auto-vectorisation capabilities, (ii) helping the compiler fuse loops, and (iii) vectorising manually using intrinsics. This bottom-up approach offers several possibilities, starting from the simplest and most portable method, and evolving toward more complex and time-consuming processes from which a better performance is expected to be obtained.

**Figure 1.**
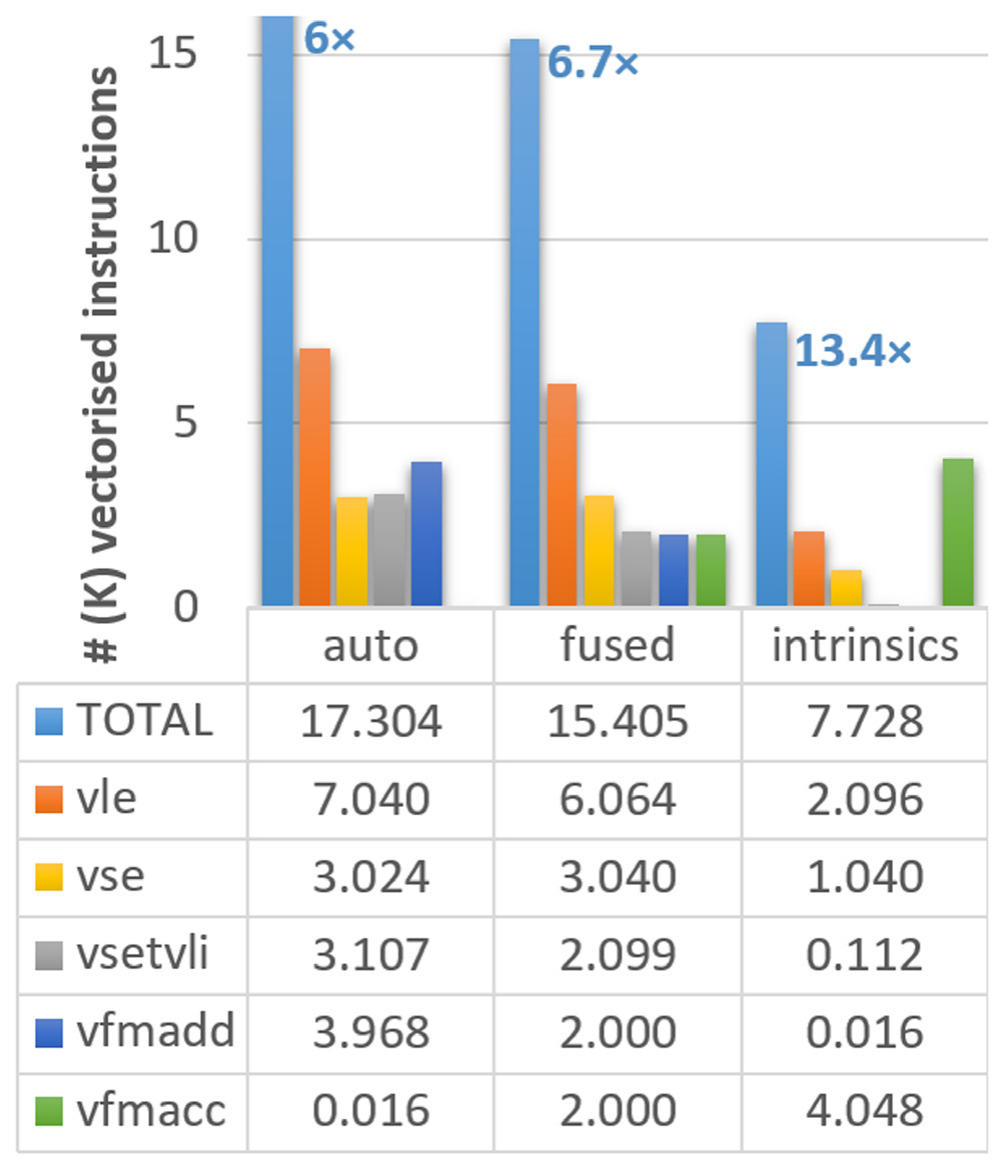
Count of vectorised instructions in the different versions of the ELPA mini-app executed on Vehave. The numbers in the bar diagram indicate the proportional reduction in vector instructions compared to our initial version with suppressed Q-stripes. Instructions are distributed by total (light blue bars), 64-bit unit-stride load (vle, orange) and save (vse, orange), settings of vector length (vsetvli, gray), and multiplication-additions (vfmadd in dark blue, vfmacc in green). The reduction in vector instructions of the differently adapted versions compared to the stripped (original) one (which counted a total of 103,784 vector instructions) is indicated over the first bar. Numbers in the graph and table are expressed in thousands.

By leveraging the compiler’s autovectorisation capabilities in the ELPA mini-app’s original version, Vehave performance analyses counted a total of 103,784 vector instructions. However, the traces show that most of the work is done with a vector length of 48 double-precision elements. This happens because ELPA was designed to divide its Q matrix into 48-element stripes. This implementation is very efficient for exploiting memory locality. However, long-vector architectures are more resistant to memory latency ^29^, and would rather benefit from a long vector length. Therefore, this striped distribution came out to be suboptimal for the VPU. Subsequently, by suppressing the Q-stripes, the algorithm starts leveraging its full vector length (256 elements per vector) without the limitation of 48 elements, and the vector instructions are reduced by a factor of 6 *×* compared to the original version (from 103,784 to 17,304 vector instructions). We should note that since our Vehave version was shipped with the v1.0 of the EPI compiler, we could verify that the outcomes from the Fortran and C versions were equivalent at this stage. The number of vector instructions for each code version is presented in Figure 1, where the different instruction types are noted in the caption.

Upon inspection of the compilation output, we still identify several loops in which the compiler is not able to reuse loaded vectors, so memory accesses are replicated in a sub-optimal manner. Therefore, our next strategy consisted of assisting the compiler by identifying loops going through the same variables and combining (fusing) them in the code. This strategy allows us to reduce our memory accesses ( *vle* calls in Figure 1) from 7,040 to 6,064. With these changes, the total number of instructions improves by a factor of 6.7 *×*, an additional 11% with respect to the previous version (from 17,304 to 15,405). Moreover, this strategy is expected to improve the performance of any vectorising compiler.

Our third and more complex method was using intrinsics, which, in some circumstances, can outperform the simple autovectorisation porting approach. The use of intrinsics allows the expansion of vectorisation, creating a pipeline of vector instructions in an outer loop, further reducing the number of memory accesses. This strategy reduces the vector instructions to 9,499, achieving an improvement of 13.4 *×* compared to the initial version.

However, not all instructions have the same computational cost, so converting from the vector instructions counters to computing time (or speed up) may not be straightforward. We observe that our modifications managed to substantially reduce the number of memory accesses (loads ( *vle*) and store ( *vse*)), which are among the most computationally costly instructions. In fact, almost 2/3 of the *vle* and *vse* have been suppressed in the version with intrinsics. On the other hand, the accumulated number of fused multiply-add instructions ( *vfmadd* and *vfmacc* are equivalent) remains almost constant. The settings of vector length ( *vsetvli*) are also progressively reduced; however, their overall contribution to the total number of cycles is almost negligible compared to other instructions. In summary, we can conclude that our results show that all instruction types are being consistently reduced, guaranteeing the improved efficiency of the new implementation.

**Figure 2.**
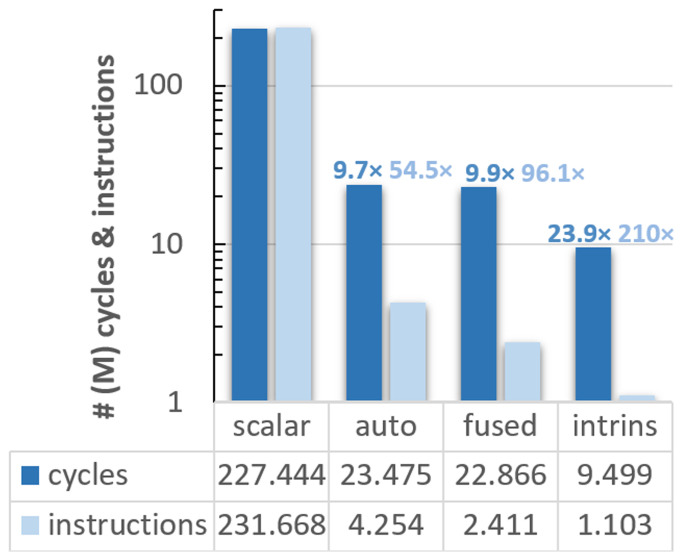
Count of cycles (dark blue bars) and instructions (light blue) for the different vectorised versions of the ELPA mini-app executed on the FPGA system. The speed-up of the differently adapted versions compared to the scalar ones is indicated over the bars, following the same colour code. The y-axis is presented in a logarithmic scale, and all numbers are expressed in millions. PAPI counters were used for these implementations.

After our preliminary study with Vehave, we used an experimental platform to evaluate the RISC-V VPU, which is composed of an FPGA, and a host-x86 server used to program and communicate with it. In addition to measuring hardware counters such as the number of vector instructions, running on the FPGA allows one to obtain cycle-accurate time measurements. This metric enables a more straight-forward interpretation of how much the code’s efficiency was improved at each implementation. The outcomes of our analyses are presented in Figure 2.

In this case, the scalar version exhibits almost a one-to-one ratio between cycles (227,443,896) and instructions (231,667,638). Compared to the scalar version, the autovectorised one reduces the cycles and instructions by a factor of 9.7 *×* and 54.5 *×*, respectively. The efficiency was further improved in the version with fused loops, obtaining an overall speedup of 9.9 *×* in cycles and 96.1 *×* in instructions, and 23.9 *×* and 210 *×*, respectively, using intrinsics.

## Conclusions

In this short communication, we describe our efforts to inform the implementation of new (post)-exascale HPC systems based on RISC-V. Our porting is centred around the ELPA eigensolver, a library used by many of the most widely-used *ab initio* electronic structure codes. Our porting work was done on the RISC-V core with a VPU developed at the Barcelona Supercomputing Center (BSC) within the framework of the European Processor Initiative (EPI) ^30^.

The manuscript summarises the iterative steps for improving the performance of a complex HPC library leveraging a RISC-V-based VPU prototype. Vectorisation of the kernel was achieved by (i) auto-vectorisation, (ii) fusing similar loops, and (iii) using intrinsics. This progressive approach offers several possibilities, from the most straightforward and portable approaches to the more complicated ones. The performance improvements are obtained with minimal intervention to the original code since we rely in the first instance on the autovectorisation features of the compiler. In addition to that, our vectorised code using intrinsics did not require us to implement loop unrollings, which would have increased the complexity of the code. Moreover, when relying on compiler autovectorisation, the code remains a plain C (or Fortran) code annotated with pragmas, so performance can be achieved without adding calls to external kernels, maintaining the code clean and portable. We should remark that one of the main particularities of this platform is that vector loads and stores are designed to bypass the (32-kByte) L1 cache. This architectural choice prevents large vector data, which can often exceed the cache's capacity, from polluting it content. Instead, this data is routed directly to the (larger, 1 MB) L2 cache. This design significantly reduces the complexity of managing the cache optimization, which is often one of the trickiest aspects of porting to new architectures. The platform's memory hierarchy supports faster and more predictable data access patterns, particularly in high-performance computations that involve large matrices or vectors. This characteristic allowed us to focus more on maximizing vectorization and parallelism, rather than needing to re-engineer data movement strategies between memory and cache. Our experience highlights how modern architectures, such as this one, can simplify the process of porting highly optimized kernels. Another important benefit of this architecture is the flexibility of the variable vector length, which allowed us to avoid the problem of dealing with the tails of the arrays, reducing the complexity of the codes. Our study implements standard vectorisation techniques that are widely applicable across various architectures. The RISC-V vector extension's flexibility, particularly its support for varying vector lengths, allowed us to explore long-vector implementations effectively. This characteristic is not unique to our platform, and similar capabilities are present in other architectures supporting long-vector processing, such as the NEC SX-Aurora TSUBASA. Moreover, the new v1.0 of our V-extension will allow the compilation of codes in both Fortran and C, so future porting of Fortran code will be more straightforward when the updated hardware is available.

We should note that the ELPA library has a patterned file that can be used to create specific kernels for new architectures. Therefore, while we will focus on a specific kernel, porting can be further replicated throughout the library following an analogous procedure. The experience gained provides practical guidance for other codes and architectures. So far, the performance of ELPA has been proven to be excellent for large matrices when GPUs are used, however, the CPU version has better performance for smaller problems. With the inclusion of vector architecture in a heterogeneous HPC system, the crossover point where the GPUs outperform could be potentially shifted toward larger matrix sizes. Ideally, one could imagine a homogeneous system made of EPAC-VEC where all performance improvement is delivered by the vector unit, making the development and maintance effort of the code simpler. In addition, our mini-app-based model represents a pragmatic, user-friendly approach to facilitate co-design efforts, cooperatively in fine-tuning the software and hardware components. The outcomes of these efforts will contribute significantly to advancing the porting of *ab initio* computational materials and molecular science codes - one of the most relevant families of applications with many users in the HPC community - to (post-)exascale hardware architectures developed in the EU. Furthermore, this evaluation serves as valuable feedback for hardware designers, system integrators and engineers actively involved in compilers for the systems.

## Software availability

All the code developed for this work has been openly stored in Zenodo, https://doi.org/10.5281/zenodo.12665268 ^31^, while the project is also openly available on our institutional BSC Materials Science group GitLab https://gitlab.bsc.es/material-science/elpa2riscv-public. The code is published under GNU LGPLv3 license: https://www.gnu.org/licenses/lgpl-3.0.html. The ELPA library is also open-source and can be obtained from https://elpa.mpcdf.mpg.de/. The specification of the RISC-V "V" extension v0.7.1 used in this work can be openly accessed at https://github.com/riscv/riscv-v-spec/releases/tag/0.7.1, under a Creative Commons Attribution 4.0 International Public License. The intrinsics used with the EPI compiler can also be accessed at https://admin.hca.bsc.es/epi/ftp/doc/intrinsics/EPI-0.7/epi-intrinsics.html.
