# Supplementary material for: Co-designing ab initio electronic structure methods on a RISC-V vector architecture
Source: Open Res Eur. 2025 Sep 9;4:165. Originally published 2024 Aug 5. [Version 4] doi: 10.12688/openreseurope.18321.4 (PMC11358678; doi:10.12688/openreseurope.18321.4)
Supplement: Peer review history

# Reviewer report: Reviewer response for version 4

**DOI:** 10.21956/openreseurope.21575.r59769

**Published:** 2025-09-23

**Authors:** Keichi Takahashi

**Affiliations:** 1 D3 Center, University of Osaka, Osaka, Japan

The authors have addressed all of my concerns and questions in their revised manuscript and response. I especially appreciated the additional analysis of optimality using hardware counters.

Is the study design appropriate and does the work have academic merit?

Yes

Is the work clearly and accurately presented and does it cite the current literature?

Yes

If applicable, is the statistical analysis and its interpretation appropriate?

Yes

Are all the source data underlying the results available to ensure full reproducibility?

Yes

Are the conclusions drawn adequately supported by the results?

Yes

Are sufficient details of methods and analysis provided to allow replication by others?

Yes

Reviewer Expertise:

HPC, vector processors, GPU

I confirm that I have read this submission and believe that I have an appropriate level of expertise to confirm that it is of an acceptable scientific standard.

# Reviewer report: Reviewer response for version 3

**DOI:** 10.21956/openreseurope.20502.r47178

**Published:** 2024-12-10

**Authors:** Keichi Takahashi

**Affiliations:** 1 D3 Center, University of Osaka, Osaka, Japan

This paper presents the porting effort of an eigensolver kernel to a RISC-V processor prototype targeting post-exascale computing. The kernel is extracted from the ELPA eigensolver library, which is used by various *ab initio* electron structure codes. The target processor, EPAC-VEC, features a vector processing unit with 16,384-bit wide vector registers. The authors employ a progressive approach for vectorization: (1) auto-vectorization, (2) loop fusion, and (3) the use of  intrinsics, where performance is improved at the cost of lower portability and higher implementation effort. The final version resulted in 13.4x fewer vector instructions and 23.9x fewer clock cycles.

- What is the optimality of the mini-app after porting? The paper demonstrates improvements over the initial version of the mini-app, but does not show whether the final version fully exploits the potential of the EPAC-VEC processor. I suggest adding an assessment of the optimality, such as a roofline analysis and/or vectorization rate.
- Although the paper is entitled "Co-designing", it assumes the hardware architecture as given and only describes a unidirectional effort: adapting a software to the target platform. I suggest clarifying  what insights or recommendations for the hardware designers arise based on the results of this paper.
- The employed progressive approach is a widely adopted when vectorizing applications. I suggest highlighting the porting techniques that are specific to the RISC-V vector extension or EPAC-VEC, if there are any.
- I could not follow the discussion on cache in the conclusion: "...one of the main particularities of this platform is that vector loads and stores do not pollute the cache." Does this mean all vector loads/stores bypass the cache? I suggest describing the cache design of EPAC-VEC earlier, or removing this discussion from the conclusion.

Is the study design appropriate and does the work have academic merit?

Yes

Is the work clearly and accurately presented and does it cite the current literature?

Yes

If applicable, is the statistical analysis and its interpretation appropriate?

Yes

Are all the source data underlying the results available to ensure full reproducibility?

Yes

Are the conclusions drawn adequately supported by the results?

Yes

Are sufficient details of methods and analysis provided to allow replication by others?

Yes

Reviewer Expertise:

HPC, vector processors, GPU

I confirm that I have read this submission and believe that I have an appropriate level of expertise to confirm that it is of an acceptable scientific standard, however I have significant reservations, as outlined above.

# Response

**Published:** 2025-09-04

**Authors:** julio gutierrez

We are very grateful for the referee's careful reading and constructive suggestions. Although we received this last review report once the paper had already been formally accepted, we believe the additional comments offer valuable insights that can strengthen the final version of the manuscript. In the spirit of scientific discussion, we have addressed his feedback by updating the conclusions. We hope these revisions further enhance the quality and clarity of the paper.

**Comment#1:** **What is the optimality of the mini-app after porting? The paper demonstrates improvements over the initial version of the mini-app, but does not show whether the final version fully exploits the potential of the EPAC-VEC processor. I suggest adding an assessment of the optimality, such as a roofline analysis and/or vectorization rate.**

**Response:** We thank the reviewer for this insightful suggestion. We fully agree that assessing the degree of optimality is important to evaluate how effectively the application exploits the architecture. However, our current work is based on an FPGA implementation of the EPAC-VEC processor, where performance metrics such as peak frequency or memory bandwidth are not yet stable or representative of a finalized platform. This makes traditional roofline models or absolute performance comparisons less meaningful at this stage.

 That said, we have conducted a preliminary analysis based on hardware counters. The mini-app achieved full vectorisation, with no tail effects, and the measured cycles per vector instruction (~36.4) closely match the expected latency for vector fused multiply-add operations (~35 cycles for 256 double-precision elements), which dominate the workload. This suggests the application is already utilizing the vector unit efficiently and approaching the latency-limited performance of the current implementation.

 A more complete evaluation of performance relative to architectural ceilings, including roofline analysis, will be more appropriate once the architecture matures into an ASIC form with well-defined performance bounds. We view this as an important direction for future work.

**Comment#2: Although the paper is entitled "Co-designing", it assumes the hardware architecture as given and only describes a unidirectional effort: adapting a software to the target platform. I suggest clarifying what insights or recommendations for the hardware designers arise based on the results of this paper.**

**Response:** The performance metrics obtained served as empirical data that can inform both compiler optimisations and hardware design decisions. This collaboration led to enhancements in the compiler's ability to optimise code for the RISC-V vector architecture, thereby improving overall system performance. Such iterative refinement between software requirements and compiler capabilities exemplifies the co-design paradigm, where insights from software adaptation inform and enhance compiler and hardware design. This feedback loop is essential in co-design, enabling hardware architects and compiler developers to tailor future architectures and tools to better accommodate the computational patterns observed in scientific applications.

**Comment#3: The employed progressive approach is a widely adopted when vectorizing applications. I suggest highlighting the porting techniques that are specific to the RISC-V vector extension or EPAC-VEC, if there are any.**

**Response:** Our study implements standard vectorisation techniques that are widely applicable across various architectures. The RISC-V vector extension's flexibility, particularly its support for varying vector lengths, allowed us to explore long-vector implementations effectively. The RISC-V vector extension's flexible design facilitates efficient vectorisation, particularly due to its support for varying vector lengths, enabling the exploration of long-vector implementations without necessitating architecture-specific techniques. This characteristic is not unique to RISC-V, and similar capabilities are present in other architectures, such as the NEC SX-Aurora TSUBASA. Therefore, the porting techniques we employed are not exclusive to RISC-V but are applicable to any architecture that supports long-vector processing. 

**Comment#4: I could not follow the discussion on cache in the conclusion: "...one of the main particularities of this platform is that vector loads and stores do not pollute the cache." Does this mean all vector loads/stores bypass the cache? I suggest describing the cache design of EPAC-VEC earlier, or removing this discussion from the conclusion.**

**Response:** Vector memory accesses bypass L1 cache by default. L1 cache size is 32 kBytes: Caching the data of a few vector instructions would wipe the whole content of the cache, so it is not convenient for the scalar core. L2 cache size is 1 MB, and it is also used as a cache for data involved in vector instructions. The conclusions section has been updated to include these last two clarifications.

# Reviewer report: Reviewer response for version 2

**DOI:** 10.21956/openreseurope.20353.r45574

**Published:** 2024-11-05

**Authors:** William Dawson

**Affiliations:** 1 RIKEN Center for Computational Science, Kobe, Japan

The response and updated manuscript thoroughly covers the concerns I had with the initial draft. I especially think that the new conclusion and various added pieces addressed my main issue about a lack of broad conclusions. Therefore I recommend this for indexing.

 Regarding the license: now the text still says GPLv3, and the repository has the GPLv3 license. But my impression was that both would ideally be LGPLv3, since that is the license of ELPA.

 If the authors decide to make further revisions, I have a few additional small comments about the style:

 * Page 3: "Density Functional theory" - theory should be capitalized

 * Page 3: cubic scalability -> cubic scaling growth in computational costs

 * Page 4: break up the long paragraph into several smaller ones

 * Page 4: "serve both to different purposes" -> "serve different purposes"

 * Page 6: "with a minimal intervention" -> "with minimal intervention"

 * Page 6: "did not require to" -> "did not require us to"

 * Page 6: "the CPU version outperforms" -> "the CPU version has better performance"

 * Page 6: "more users in the HPC" -> "many users in the HPC"

 * The link to "https://gitlab.bsc.es/material-science/elpa2riscv-public" is broken, containing an extra space before material

Is the study design appropriate and does the work have academic merit?

Yes

Is the work clearly and accurately presented and does it cite the current literature?

Yes

If applicable, is the statistical analysis and its interpretation appropriate?

Yes

Are all the source data underlying the results available to ensure full reproducibility?

Yes

Are the conclusions drawn adequately supported by the results?

Yes

Are sufficient details of methods and analysis provided to allow replication by others?

Yes

Reviewer Expertise:

Electronic structure calculations, high performance computing.

I confirm that I have read this submission and believe that I have an appropriate level of expertise to confirm that it is of an acceptable scientific standard.

# Response

**Published:** 2024-11-12

**Authors:** julio gutierrez

We much appreciate the comments provided again by the referee. Although the license was updated to LGPLv3 in the previous revision, this change was only visible in the repository and was not correctly indicated in the manuscript. We have fixed this and the link to our GitLab. We have also implemented the additional wording suggestions.

# Reviewer report: Reviewer response for version 1

**DOI:** 10.21956/openreseurope.19799.r43456

**Published:** 2024-09-04

**Authors:** William Dawson

**Affiliations:** 1 RIKEN Center for Computational Science, Kobe, Japan

In this paper, Torres et al. describe some early experience porting the ELPA eigenvalue solver library to a RISC-V platform featuring a wide vector unit (as part of the European Processor Initiative). They analyze how efficient the compiler generated code is, and how it can be improved through a sequence of tuning steps.

 I appreciated the opportunity to read the paper. This is a valuable article for the community, as they are targeting what may be a very relevant platform in the future. The article is cross disciplinary, yet very approachable and the main points are easy to understand. Given its forward-looking nature and narrow scope, it seems appropriate for a brief report.

 The main weakness of this paper is that the authors don’t provide any broad, transferable results from this data. If I was tasked with the porting ELPA to a production ready RISC-V supercomputer (or to any new machine), I would do exactly as the authors did: I would consider what compiler flags might be optimal, I would manually fuse loops, I would write intrinsic vectorization instructions, etc. Thus, this paper only describes the basic state-of-the-art, instead of going one step further and really evaluating the platform and creating new knowledge.

 I recommend that the authors rewrite the conclusion section, which wastes half a page repeating information that is fresh in the mind of the reader of this short report. Instead, I would like the authors to make broad recommendations from their data such as: how should developers prepare their code for the prospect of being ported to this platform, what weaknesses exist in the current compiler toolchains for this system, what other codes should be evaluated based on the weaknesses found here, should we expect this platform to perform well in comparison to other platforms for ELPA if optimization is done properly, if a supercomputer is built on this platforms and runs realistic workflows will this kernel obtain peak performance in this kernel, etc. These are just suggestions, not a to do list; I think the authors will know best which broad conclusions are best justified by the data.

  As you note, the authors of ELPA made extensive efforts to specialize for previous platforms, so what’s new about the porting experience for this platform? (or maybe nothing is, and your conclusion is that this platform works great if you use standard techniques). This kind of analysis will really allow the authors to impact the community by leveraging their experience and expertise.

 Two other broad criticisms I have are:

1. It is not clear to me why the authors use both the Vehave emulator and the FPGA implementation. What are the strengths of the emulator in this regard? Why not just use the (cycle accurate) FPGA? Are the systems tested the same in both cases? Maybe a broad takeaway from this paper could be about which tools to use.
2. The conversion of the code from Fortran to C risks making this benchmark less realistic. Maybe when the Fortran compiler for this platform becomes available, the compiler would be able to do these optimizations without any problem. If you try to compile the C version you wrote on other platforms, how well does it perform against the Fortran version? Maybe your C conversion introduced artificial challenges for the compiler. I think some checks about this would make the paper more solid.

In addition to these broad recommendations, I have some other small comments which the authors might consider for improving their paper.

- In the abstract, just calling Vehave “the user-space Vehave” is unclear, you should write that it’s an emulator there.
- In the introduction, they cite a roadmap article. Since the introduction specifically talks about modular design, they may also consider citing an article describing the CECAM effort [1]
- When describing the importance of eigenvalue problems, the authors should clarify that ELPA targets the dense eigenvalue problem, to distinguish it from sparse solvers (though ELPA is used as a building block for sparse solvers).
- The authors specifically say that eigenvalue solvers take “90% of the total computational time in relatively large systems”. Since they have a specific number there, they should cite where they got it from.
- The authors should describe more specifically what they are testing on. “ELPA checkpoints for matrices with different sizes are accessible” – but what size are you using here? Do you use all the data or just one specific problem?
- I am pretty sure the data you have labeled “auto” is after the Q-stripes are suppressed, but if you could explicitly note this point, I think that’d be clearer.
- For Fig. 2, it could help to repeat the color scheme of Fig. 1 instead of using two shades of blue. I also liked that in Fig. 1 you had a patch with the colors in the table, you should do that in Fig. 2 as well.
- In the paper you claim the code is published a GPL, but on the repository it’s LGPL (as it should be so that code can be backported to ELPA).
- Reference 24 mistakenly lists “Departament Computadors” as an author. 

Some grammatical / spelling / stylistic corrections I suggest:

- Fig. 2 table: clycles => “cycles”.
- Various places: vectorial instructions => “vectorized instructions” or sometimes “vector instructions” depending on the context, vectorial compiler => vectorizing compiler, etc.
- Introduction, paragraph 1: easily adapted to incorporate => more adaptable to the incorporation of
- Introduction, paragraph 1: and to perform efficiently heterogeneous computing systems. => and to perform efficiently “on” heterogeneous computing systems.
- Introduction, paragraph 2: FHI-aims, GPAW, or CP2K => FHI-aims, GPAW, “and” CP2K.
- Introduction, paragraph 2: The library can be directly implemented => The library can be directly “integrated”
- Introduction, paragraph 3: a significant advancement HPC => a significance advancement “in” HPC
- Introduction, paragraph 4: the relevance of the eigensolver => the relevance of eigensolvers
- Introduction, paragraph 4: landmark work => pioneering works
- Methodology, paragraph 2: runs on top of the RISC-V => runs on top of RISC-V
- Methodology, paragraph 2: This FPGA is used as user-defined => This FPGA is used as a user-defined.
- Methodology paragraph 2: this link => this looks weird with the superscript citations, maybe replace “this link” with “online”.
- Methodology paragraph 3: computationally cost => computational cost.
- Methodology paragraph 3: especially to the extensive => especially for the extensive
- Methodology paragraph 3: the indications to execute => the instructions to execute
- Methodology paragraph 3: associated online repositories. Here a link would be good (I know it comes at the end of the article, but it’d be convenient to have it here too).
- Results and discussion paragraph 3: The increasing performance. I don’t understand what this sentence means. I also don’t understand why you characterize your strategy as bottom-up.
- Results and discussion paragraph 4: counted for a total => counted a total.
- Results and discussion paragraph 4: without limitations of 48 elements => without the limitation of 48 elements
- Results and discussion paragraph 6: either put a comma or a colon after 9,499.
- Results and discussion paragraph 7: On the other hand, => I was confused by this part until I realized you meant the sum of vfmadd and vfmacc remains constant, not each one individually.
- Results and discussion paragraph 8: RISC-V VPU, composed of an FPGA, and a => RISC-V VPU, which is composed of an FPGA and a

I don’t have access to a RISC-V machine, but I tested out your source code in the gitlab repository on an x86 machine. I think the instructions were easy to follow, but I had some questions:

- M_REAL_SIMPLE_F – what optimizations are done here? It’s faster than  M_REAL_REFERENCE, but slower than M_REAL_SIMPLE.
- What is the difference between GENERIC and SIMPLE? Why is GENERIC slower than simple?

Is the study design appropriate and does the work have academic merit?

Yes

Is the work clearly and accurately presented and does it cite the current literature?

Yes

If applicable, is the statistical analysis and its interpretation appropriate?

Yes

Are all the source data underlying the results available to ensure full reproducibility?

Yes

Are the conclusions drawn adequately supported by the results?

Yes

Are sufficient details of methods and analysis provided to allow replication by others?

Yes

Reviewer Expertise:

Electronic structure calculations, high performance computing.

I confirm that I have read this submission and believe that I have an appropriate level of expertise to confirm that it is of an acceptable scientific standard, however I have significant reservations, as outlined above.

## References

[1] : The CECAM electronic structure library and the modular software development paradigm. *J Chem Phys* .2020;153(2) : 10.1063/5.0012901 024117 10.1063/5.0012901 32668924

# Response

**Published:** 2024-10-23

**Authors:** julio gutierrez

***Comment #1: In this paper, Torres et al. describe some early experience porting the ELPA eigenvalue solver library to a RISC-V platform featuring a wide vector unit (as part of the European Processor Initiative). They analyze how efficient the compiler generated code is, and how it can be improved through a sequence of tuning steps.** **I appreciated the opportunity to read the paper. This is a valuable article for the community, as they are targeting what may be a very relevant platform in the future. The article is cross disciplinary, yet very approachable and the main points are easy to understand. Given its forward-looking nature and narrow scope, it seems appropriate for a brief report.** **The main weakness of this paper is that the authors don’t provide any broad, transferable results from this data. If I was tasked with the porting ELPA to a production ready RISC-V supercomputer (or to any new machine), I would do exactly as the authors did: I would consider what compiler flags might be optimal, I would manually fuse loops, I would write intrinsic vectorization instructions, etc. Thus, this paper only describes the basic state-of-the-art, instead of going one step further and really evaluating the platform and creating new knowledge.***

**Answer: **We would like to referee for his thorough revision and the suggested comments and corrections, that we believe have help us to improve the manuscript substantially. The revision was made according to the referee’s requirements and we answered all points made. Individual responses are given after each comment. Before addressing the following points, we would like to clarify that the main novelty of our work lays on the use of this novel RISC-V VPU (and its associated compiler suite) to solve the eigenvalue problem. One of the main particularities of this platform is that vector loads and stores do not pollute the cache. This significantly reduces the complexity of managing the cache optimization, which is often one of the trickiest aspects of porting to new architectures. The platform's memory hierarchy supports faster and more predictable data access patterns, particularly in high-performance computations that involve large matrices or vectors. This characteristic allowed us to focus more on maximizing vectorization and parallelism, rather than needing to re-engineer data movement strategies between memory and cache. Our experience highlights how modern architectures, such as this one, can simplify the process of porting highly optimized kernels. The lessons learned—particularly the ease of porting due to efficient vector register handling—will help inform future efforts in porting scientific computing libraries to similar platforms. The reduced complexity around cache management is a particularly valuable insight that we believe will be beneficial to the broader HPC community.  

***Comment #2: I recommend that the authors rewrite the conclusion section, which wastes half a page repeating information that is fresh in the mind of the reader of this short report. Instead, I would like the authors to make broad recommendations from their data such as: how should developers prepare their code for the prospect of being ported to this platform, what weaknesses exist in the current compiler toolchains for this system, what other codes should be evaluated based on the weaknesses found here, should we expect this platform to perform well in comparison to other platforms for ELPA if optimization is done properly, if a supercomputer is built on this platforms and runs realistic workflows will this kernel obtain peak performance in this kernel, etc. These are just suggestions, not a to do list; I think the authors will know best which broad conclusions are best justified by the data.***

**Answer: **There are two main advantages in following the approach shared in this paper:

 1. The performance improvements are obtained with a minimal intervention to the original code since we rely in the first instance on the autovectorisation features of the compiler. In addition to that, our vectorised code using intrinsics did not require to implement loop unrollings, which would have increased the complexity of the code.

 2. When relying on compiler autovectorization, the code remains a plain C (or Fortran) code annotated with pragmas. This means that performance can be achieved without adding calls to external kernels (e.g., no CUDA dependencies) thus maintaining the code clean and portable. Another important benefit of this architecture is the flexibility of the variable vector length, which allowed us to avoid the problem of dealing with the tails of the arrays, reducing the complexity of the codes.

 So far, the performance of ELPA has been proven to be excellent for large matrices when GPUs are used, however, the CPU version outperforms for smaller problems. With the inclusion of vector architecture in a heterogeneous HPC system, the crossover point where the GPUs outperform could be potentially shifted toward larger matrix sizes. Ideally, one could imagine a homogeneous system made of EPAC-VEC where all performance improvement is delivered by the vector unit, making the development and maintance effort of the code simpler. 

 We have tested EPAC-VEC for other scientific software applications such as Alya, a fluid dynamic code developed in Fortran and we demonstrated that a significant speed-up (up to 7×) can be achieved with code modifications that leave the code portable and deliver performance improvements also on Intel x86 architectures (while leveraging AVX512 SIMD extension by Intel). [Blancafort, Marc, et al. "Exploiting long vectors with a CFD code: a co-design show case." 2024 IEEE International Parallel and Distributed Processing Symposium (IPDPS)]. The EPI team also proved that the EPAC-VEC architecture enables significant performance improvement when dealing with non-dense linear algebra workloads. In this paper, for example, Sparse-matrix algebra and kernels are considered [Vizcaino, Pablo, et al. "Short reasons for long vectors in HPC CPUs: a study based on RISC-V." Proceedings of the SC'23 Workshops of The International Conference on High Performance Computing, Network, Storage, and Analysis. 2023. https://arxiv.org/abs/2309.06865]. However, for workloads such as graph analytics or FFT computation a previous rework of the algorithm for accessing the data is required. Still, this work can be encapsulated into “vectorised libraries” and made available to the user, making the process of porting smoother. A key role in the process of testing and adopting the EPAC-VEC architecture is played by the tools used for analysing the vectorization achieved. The mental scheme of the scientist can create conceptual barriers when analysing the code. For this reasons a set of tools have been developed for analysing vectorization of the code and understand potential for vectorization [Vizcaino, Pablo, et al. "RAVE: RISC-V Analyzer of Vector Executions, a QEMU tracing plugin." arXiv preprint arXiv:2409.13639 (2024). https://arxiv.org/abs/2409.13639] The manuscript has been revised in several key areas.

 The summary part included in the conclusions section has been shortened. Additionally, various comments regarding outlook aspects and specific details about the platform have been incorporated into the text. Furthermore, the description of the Alya use case has been expanded in the introduction.  

***Comment #3: As you note, the authors of ELPA made extensive efforts to specialize for previous platforms, so what’s new about the porting experience for this platform? (or maybe nothing is, and your conclusion is that this platform works great if you use standard techniques). This kind of analysis will really allow the authors to impact the community by leveraging their experience and expertise.***

**Answer: **Each new platform presents its own set of challenges and opportunities, particularly when it comes to fully leveraging the architecture’s unique characteristics. As it was mentioned before in the response, one of the most unique features of this platform is that vector loads and stores do not pollute the cache, which simplifies managing the cache optimizations, being these particularly critical in computations involving large matrices and/or vectors. This characteristic would allow the developer to focus more on maximizing vectorization and parallelism, rather than re-engineer data movement strategies between memory and cache. This has been highlighted in the conclusions section.

***Comment #4:** **Two other broad criticisms I have are:***

1. ***It is not clear to me why the authors use both the Vehave emulator and the FPGA implementation. What are the strengths of the emulator in this regard? Why not just use the (cycle accurate) FPGA? Are the systems tested the same in both cases? Maybe a broad takeaway from this paper could be about which tools to use.***
2. ***The conversion of the code from Fortran to C risks making this benchmark less realistic. Maybe when the Fortran compiler for this platform becomes available, the compiler would be able to do these optimizations without any problem. If you try to compile the C version you wrote on other platforms, how well does it perform against the Fortran version? Maybe your C conversion introduced artificial challenges for the compiler. I think some checks about this would make the paper more solid.***

**Answers:**

 1. The Vehave emulator and the FPGA implementation serve both to different purposes and offers unique advantages. The Vehave emulator is primarily used for flexibility during the early stages of development. It allows for quick iteration, debugging, and testing of different configurations without needing to reconfigure hardware. This is particularly useful when exploring architectural optimisations or validating functionalities under varying conditions. The emulator also provides a high-level view of performance trends and functionality before transitioning to hardware-based testing. On the other hand, the FPGA implementation offers cycle-accurate performance and power consumption data. While emulators are useful for early testing and development, FPGAs enable us to test the design in real-world conditions with accurate timing, ensuring that the system meets performance targets in a hardware environment. Furthermore, the FPGA validates that our design operates as expected when mapped onto a physical implementation. This clarification has been added to the methods section.

 2. Regarding the porting from Fortran to C, and connecting also with 4.1, we should clarify that at the time this work was carried out, we could work on Vehave using the latest v1.0 implementation of EPI, while the FPGA was on the previous v0.7.1. This new v1.0 of the V-extension allowed the compilation of codes in both Fortran and C. While the code porting in this case was extremely simple and straightforward, we also used the Vehave on v1.0 to extract traces and verify that both codes were equivalent. Whereas, of course, the binaries produced from the two codes where different, the number and type of vector instructions produced by both versions were proven to be analogous. This point has been clarified in the text.

 Also, it is important to note that the original distribution of ELPA already contains two types of kernels: generic kernels written in Fortran and optimized kernels written in C. The C kernels are highly optimized for specific architectures and are used to achieve maximum performance on those platforms. The Fortran kernels, on the other hand, are generic and designed to be portable across different systems. Our implementation is based on a generic Fortran kernel, however, it is important to clarify these ones are not used by default in modern machines. On current architectures, the default kernels are specifically optimized for the hardware and are written in C. These optimized C kernels are designed to leverage platform-specific features for improved performance, which aligns with the approach we followed in our work.  

***Comment #5:** **In addition to these broad recommendations, I have some other small comments which the authors might consider for improving their paper.***

- ***In the abstract, just calling Vehave “the user-space Vehave” is unclear, you should write that it’s an emulator there.***
- ***In the introduction, they cite a roadmap article. Since the introduction specifically talks about modular design, they may also consider citing an article describing the CECAM effort [Oliveira MJT, Papior N, Pouillon Y, et al.: The CECAM electronic structure library and the modular software development paradigm. J Chem Phys. 2020; 153 (2): 024117]***
- ***When describing the importance of eigenvalue problems, the authors should clarify that ELPA targets the dense eigenvalue problem, to distinguish it from sparse solvers (though ELPA is used as a building block for sparse solvers).***
- ***The authors specifically say that eigenvalue solvers take “90% of the total computational time in relatively large systems”. Since they have a specific number there, they should cite where they got it from.***
- ***The authors should describe more specifically what they are testing on. “ELPA checkpoints for matrices with different sizes are accessible” – but what size are you using here? Do you use all the data or just one specific problem?***
- ***I am pretty sure the data you have labeled “auto” is after the Q-stripes are suppressed, but if you could explicitly note this point, I think that’d be clearer.***
- ***For Fig. 2, it could help to repeat the color scheme of Fig. 1 instead of using two shades of blue. I also liked that in Fig. 1 you had a patch with the colors in the table, you should do that in Fig. 2 as well.***
- ***In the paper you claim the code is published a GPL, but on the repository it’s LGPL (as it should be so that code can be backported to ELPA).***
- ***Reference 24 mistakenly lists “Departament Computadors” as an author. ***

**Answer: **We thank the reviewer for all these corrections and suggestions of improvements. All the indicated corrections have been implemented in the text. In addition, we would like to clarify that:

- The estimation of the relative computational time spent in the eigensolver is mostly based on our extensive user experience, going across several electronic structure codes over more than a decade. This has been clarified in the text.
- ELPA is shipped along with a variety of mini-apps to test the different kernels and precision levels on several types of matrices. We were interested in the one that gets the eigenvectors of a real double-precision matrix using the 2-stage method solver for a random matrix. We saved different checkpoints of the application at the entrance of the function *trans_ev_tridi_to_band*. Each checkpoint is labeled with the matrix size, the block size, and the number of eigenvectors that are computed. Note that we have vectorised our code in the direction of the number of eigenvectors and it is very sensitive to this value.

For the sake of correctness, we tested all the kernels of our code using Vehave, using every available checkpoint. To test the vector behavior, we focused on our most relevant cases, with large matrix sizes and a number of eigenvectors greater than the vector length. The default checkpoint file is a matrix of 1024 elements, 256 eigenvectors and a blocksize of 32 elements. This has been clarified at the end of the methods section.

- We agree that not mentioning that our reference version does not have Q-stripes could be misleading. This information has been clarified in Fig.1’s caption.
- Figure 1, the blue bars are used represented the total number of instructions for each code version, while other colours are used for partial contributions. Following that same reasoning, blue is used for the bars in Figure 2, which show also total number of cycles and instructions. We believe that using an identical colour scheme, although more visual, could also confuse the reader. In any case, the colours have been updated with brighter tones, the patches in the table have also been added to Figure 2, and the typo identified in the next block of comments has been corrected.
- Our repositories have been updated to LGPL-v3.

***  Comment #6: Some grammatical / spelling / stylistic corrections I suggest:***

- ***Fig. 2 table: clycles => “cycles”.***
- ***Various places: vectorial instructions => “vectorized instructions” or sometimes “vector instructions” depending on the context, vectorial compiler => vectorizing compiler, etc.***
- ***Introduction, paragraph 1: easily adapted to incorporate => more adaptable to the incorporation of***
- ***Introduction, paragraph 1: and to perform efficiently heterogeneous computing systems. => and to perform efficiently “on” heterogeneous computing systems.***
- ***Introduction, paragraph 2: FHI-aims, GPAW, or CP2K => FHI-aims, GPAW, “and” CP2K.***
- ***Introduction, paragraph 2: The library can be directly implemented => The library can be directly “integrated”***
- ***Introduction, paragraph 3: a significant advancement HPC => a significance advancement “in” HPC***
- ***Introduction, paragraph 4: the relevance of the eigensolver => the relevance of eigensolvers***
- ***Introduction, paragraph 4: landmark work => pioneering works***
- ***Methodology, paragraph 2: runs on top of the RISC-V => runs on top of RISC-V***
- ***Methodology, paragraph 2: This FPGA is used as user-defined => This FPGA is used as a user-defined.***
- ***Methodology paragraph 2: this link => this looks weird with the superscript citations, maybe replace “this link” with “online”.***
- ***Methodology paragraph 3: computationally cost => computational cost.***
- ***Methodology paragraph 3: especially to the extensive => especially for the extensive***
- ***Methodology paragraph 3: the indications to execute => the instructions to execute***
- ***Methodology paragraph 3: associated online repositories. Here a link would be good (I know it comes at the end of the article, but it’d be convenient to have it here too).***
- ***Results and discussion paragraph 3: The increasing performance. I don’t understand what this sentence means. I also don’t understand why you characterize your strategy as bottom-up.***
- ***Results and discussion paragraph 4: counted for a total => counted a total.***
- ***Results and discussion paragraph 4: without limitations of 48 elements => without the limitation of 48 elements***
- ***Results and discussion paragraph 6: either put a comma or a colon after 9,499.***
- ***Results and discussion paragraph 7: On the other hand, => I was confused by this part until I realized you meant the sum of vfmadd and vfmacc remains constant, not each one individually.***
- ***Results and discussion paragraph 8: RISC-V VPU, composed of an FPGA, and a => RISC-V VPU, which is composed of an FPGA and a***

**Answer: **We thank the reviewer for all these corrections and suggestions of improvements. The suggested corrections have been incorporated to the text.

- The link to the mini-apps repository has been added.
- We would like to clarify that we characterized our strategy as bottom-up because it starts with the least effort and gradually increases in complexity. This approach is practical because it allows for incremental improvements and ensures that simpler, more portable solutions are considered before resorting to more complex and time-consuming methods. The corresponding paragraph has been revised to highlight this aspect.

*** Comment #7: I don’t have access to a RISC-V machine, but I tested out your source code in the gitlab repository on an x86 machine. I think the instructions were easy to follow, but I had some questions:***

- ***M_REAL_SIMPLE_F – what optimizations are done here? It’s faster than M_REAL_REFERENCE, but slower than M_REAL_SIMPLE.***
- ***What is the difference between GENERIC and SIMPLE? Why is GENERIC slower than simple?***

**Answers:**

- M_REAL_SIMPLE is the same kernel as M_REAL_REFERENCE, the only difference is that we disabled compiler vectorization in the reference version. M_REAL_SIMPLE_F is the original ELPA code, written in Fortran. In our case, the C compiler seems to be doing a better job than the Fortran compiler.
- The generic kernel includes several loop unrolls designed to enhance instruction pipelining. This delivers good results when the optimisation levels are reduced and vectorisation is disabled. Our compiler does better optimizations with the simple kernel.

# Reviewer report: Reviewer response for version 1

**DOI:** 10.21956/openreseurope.19799.r42938

**Published:** 2024-08-28

**Authors:** Roman Wyrzykowski

**Affiliations:** 1 Czestochowa University of Technology, Częstochowa, Poland

**Summary of the article**

 This paper deals with porting ab initio electronic structure applications to an innovative RISC-V prototype platform. This platform's distinguished feature is a wide Vector Processing Unit (VPU) that can process vectors up to 256 double-precision elements, 32 times larger than current SIMD architectures. In these widely used HPC codes, solving the eigenvalue problem is often the critical computational bottleneck, consuming even up to 90% of the total computational time. Focusing on a mini-application extracted from the ELPA eigensolver library, the de facto standard in the domain, the authors systematically optimize performance targeting RISC-V-based technologies fully developed within the EU. 

**Details**

 In the systematic optimization methodology, the authors use three strategies to leverage vector instructions and increase computational efficiency, namely, (i) auto-vectorization, (ii) loop fusion, and (iii) manual vectorization with intrinsic. What is important is that performance is evaluated not only with software emulation but also on an FPGA-based hardware platform, showing outstanding improvements in instruction count and execution cycles. 

 Among the primary indicators of performance improvements are the following:

 1. For the ELPA mini-app executed on the FPGA platform, the auto-vectorization reduces instructions and cycles by a factor of respectively 54.5x and 9.7x, compared to the scalar version.

 2. The performance was finally improved in the version with intrinsics, obtaining a speedup of 210x in instructions, and 23.9x in cycles.

 The reviewed research demonstrates the massive potential of the co-design approach and RISC-V open architecture for developing future HPC hardware and compilers, which can achieve unprecedented application performance and computational efficiency. This work makes a clear contribution to European strategy in the HPC domain and promotes technologies fully developed within the EU.       

****

Is the study design appropriate and does the work have academic merit?

Yes

Is the work clearly and accurately presented and does it cite the current literature?

Yes

If applicable, is the statistical analysis and its interpretation appropriate?

Yes

Are all the source data underlying the results available to ensure full reproducibility?

Yes

Are the conclusions drawn adequately supported by the results?

Yes

Are sufficient details of methods and analysis provided to allow replication by others?

Yes

Reviewer Expertise:

HPC, computing architectures, parallel algorithns and applications

I confirm that I have read this submission and believe that I have an appropriate level of expertise to confirm that it is of an acceptable scientific standard.

# Response

**Published:** 2024-10-23

**Authors:** julio gutierrez

We are very grateful for your positive feedback on our paper. Your thorough review and encouraging comments are greatly appreciated. We are pleased to know that our work has met your expectations, and we value the time and effort you have invested in reviewing our manuscript.

# Reviewer report: Reviewer response for version 1

**DOI:** 10.21956/openreseurope.19799.r42945

**Published:** 2024-08-28

**Authors:** Ahmed Kamaleldin

**Affiliations:** 1 Technische Universitat Dresden, Dresden, Germany

This article presents a method to port the ELPA eigensolver to the RSIC-V based vector processor architecture (EPAC-VEC). The architecture is part of the EPI project, it can handle vectors up to 256 double-precision elements. 

 The execution was performed on a user-space emulator called Vehave for RISC-V V ISA. The article proposal adapts the ELPA mini-app to run on a long vector architecture. 

 Several compiler autovectorisation techniques are investigated: 1) auto-vectorization, 2) fusing similar loops, and 3) using intrinsics.

 The evaluation shows that applying those auto-vectorization techniques can reduce the instruction counts by 13.4x and the total number of clock cycles.

 Comments: 

 1- Adding several applications (as use cases) for evaluation is recommended. 

 2- Evaluating the energy efficiency and achieved computing performance is recommended. 

 3- A comparison with other HPC vector processors could be useful for evaluation.

Is the study design appropriate and does the work have academic merit?

Yes

Is the work clearly and accurately presented and does it cite the current literature?

Yes

If applicable, is the statistical analysis and its interpretation appropriate?

Yes

Are all the source data underlying the results available to ensure full reproducibility?

Yes

Are the conclusions drawn adequately supported by the results?

Yes

Are sufficient details of methods and analysis provided to allow replication by others?

Yes

Reviewer Expertise:

System-on-chip, Reconfigurable computing, Multi-core architecture, Heterogeneous compute systems

I confirm that I have read this submission and believe that I have an appropriate level of expertise to confirm that it is of an acceptable scientific standard.

# Response

**Published:** 2024-10-23

**Authors:** julio gutierrez

We thank the referee for his feedback on our paper. Regarding the comments provided, we would like to clarify that:

1. The ELPA library is currently used by several of the most popular electronic structure application packages, some of them cited in the manuscript. The present kernel represents a key section in the diagonalisation of a dense matrix, which is a relevant use case to many of this family of codes. While testing the performance of a full application on the VPU is not possible at the moment, this will definitely be an interesting continuation of this work, which we plan to carry out in the near future.
2. We agree that measuring energy consumption would add insight information to the evaluated technology. However, thorough energy measurements cannot be shared due to NDA constraints enforced by the EPI consortium. We can add that:
  1. The EPAC-VEC test chip is implemented using Global Foundries 22nm transistor technology, which is a fairly outdated chip technology compared with the state-of-the-art chip manufacturing technology. While the test chip development effort still represents a ground-breaking step forward for European technological horizon, the detailed power figures would be at this stage probably not representative.
  2. The EPAC-VEC test chip does not embed a memory controller (it relies on a high-speed SerDes and an external FPGA for memory and I/O operations): this would make the comparison of power drain figures difficult to compare with most state-of-the-art established technologies.
  3. RISC-V is a new, simple and modular ISA used to guarantee a lean implementation (as compared to incremental ISAs such as Intel x86 that need to guarantee support to legacy instruction), theoretically leading to a more energy efficient final solution.
  4. Since the architecture of EPAC-VEC explores a design point that is fairly extreme (using extremely wide vectors), the main goal of our work is to address programmability and performance gain delivered by this architecture.
3. NEC Aurora SX is the only large vector accelerator with an architecture comparable to the one used in EPAC-VEC. While the comparison is definitely possible, the analysis of the results should take into account silicon area considerations. NEC Aurora SX architecture in fact treats the same vector size using 12× more functional units, delivering higher performance at a significant cost in terms of area. Thus, in the interest of space and clarity of the presentation we decided to keep this comparison out of this paper and will consider it for an extension.

# Reviewer report: Reviewer response for version 1

**DOI:** 10.21956/openreseurope.19799.r42941

**Published:** 2024-08-21

**Authors:** Flavio Vella

**Affiliations:** 1 University of Trento, Trento, Italy

The document discusses the integration of **ab initio electronic structure methods** with a RISC-V vector architecture in High-Performance Computing (HPC). Ab initio calculations, particularly in Density Functional Theory (DFT), are computationally intensive, with eigenvalue problems often being the primary bottleneck, consuming up to 90% of total computational time in large systems.

 The project **focuses on porting the ELPA eigensolver library to a RISC-V prototype featuring a wide Vector Processing Unit (VPU).** This VPU can handle vectors up to 256 double-precision elements, significantly larger than those managed by conventional SIMD architectures like Intel’s AVX-512. The approach involves using a mini-app from the ELPA library to optimize performance on the RISC-V platform.

**Details** 

 Three optimization strategies were employed to reduce vector instructions and improve computational efficiency. 1) autovectorisation, 2) loop fusion, and 3)manual vectorisation using intrinsics.

 Performance was evaluated using both emulation and FPGA platforms, showing significant reductions in execution cycles and instruction counts.

 The work is positioned within the broader context of preparing ab initio methods for exascale computing, where increasing architectural heterogeneity is expected. The co-design approach provides critical feedback for the development of future HPC hardware and compilers, influencing the design of post-exascale systems. This research contributes to the EU's strategic goals in HPC, advancing the readiness of key computational codes for next-generation systems.

**Key Performance Indicators**

 Reduction in Vector Instructions. Initial implementation with autovectorization resulted in 103,784 vector instructions.

1. By suppressing the Q-stripes and leveraging the full vector length of 256 elements, vector instructions were reduced to 17,304, a 6x reduction.
2. Further optimization with loop fusion reduced the vector instructions to 15,405, a 6.7x reduction from the original.
3. Manual vectorization using intrinsics reduced the vector instructions to 9,499, achieving a 13.4x improvement over the initial version.

Performance Improvements. The scalar version required 227,443,896 cycles and 231,667,638 instructions.

1. Autovectorization reduced cycles and instructions by 9.7x and 54.5x, respectively.
2. With fused loops, there was a 9.9x reduction in cycles and a 96.1x reduction in instructions.
3. Using intrinsics, the improvements reached 23.9x in cycles and 210x in instructions compared to the scalar version.

Memory Access Reduction:

 The optimization strategies led to significant reductions in memory accesses, with a focus on the costly load (vle) and store (vse) instructions. For example, memory accesses (vle calls) were reduced from 7,040 to 6,064 after loop fusion.

Is the study design appropriate and does the work have academic merit?

Yes

Is the work clearly and accurately presented and does it cite the current literature?

Yes

If applicable, is the statistical analysis and its interpretation appropriate?

Yes

Are all the source data underlying the results available to ensure full reproducibility?

Yes

Are the conclusions drawn adequately supported by the results?

Yes

Are sufficient details of methods and analysis provided to allow replication by others?

Yes

Reviewer Expertise:

High-Performance Computing, GPU Computing, Distributed-memory systems.

I confirm that I have read this submission and believe that I have an appropriate level of expertise to confirm that it is of an acceptable scientific standard.

# Response

**Published:** 2024-10-23

**Authors:** julio gutierrez

We sincerely thank you for your positive feedback on our paper and for taking the time to thoroughly review it.
